# Simultaneous Therapy Approach: Systemic and Intratympanic Corticosteroid for Idiopathic Sudden Sensorineural Hearing Loss: A Clinical Study

**DOI:** 10.22038/ijorl.2025.89180.3984

**Published:** 2025

**Authors:** Mohsen Rajati, Imaneh Roshanzamir, Ermia Mousavi Mohammadi, Romina Tavasoli, Sadegh Jafarzadeh

**Affiliations:** 1 *Sinus and Surgical Endoscopic Research Center, School of Medicine, Mashhad University of Medical Sciences, Mashhad, Iran.*; 2 *Department of Otorhinolaryngology, School of Medicine, Mashhad University of Medical Sciences, Mashhad, Iran.*; 3 *Department of Otorhinolaryngology, School of Medicine, Mashhad University of Medical Sciences, Mashhad.*; 4 *Department of Audiology, School of Paramedical and rehabilitation Sciences, Mashhad University of Medical Sciences, Mashhad, Iran.*

**Keywords:** Sensorineural hearing loss, Sudden hearing loss, Corticosteroids, Vertigo, Tinnitus

## Abstract

**Introduction:**

Idiopathic Sudden sensorineural hearing loss (ISSNHL) can be treated with various methods. This study investigated the impact of combined systemic and intratympanic corticosteroid injections on hearing improvement and associated symptoms in patients with ISSNHL.Top of Form.

**Materials and Methods:**

The study investigated the recovery process of 73 patients with ISSNHL from Khorasan Razavi registry between the years 2022 to 2024. These patients received treatment involving both systemic and intratympanic corticosteroid.

**Results:**

One month after treatment, the study using Modified Siegel's criteria found complete, partial, and slight improvement in 30%, 14%, and 16% of patients, respectively. During this period, 24% of cases with tinnitus were completely treated, while 51% experienced partial relief. For cases with vertigo, 67% were completely treated, and 33% had partial improvement.

**Conclusion:**

Combined corticosteroid treatment is effective in approximately sixty percent of ISSNHL cases. This therapy also demonstrates success in alleviating related symptoms such as tinnitus and vertigo.

## Introduction

Idiopathic Sudden sensorineural hearing loss (ISSNHL) is characterized by a rapid onset of hearing loss upon waking up or a gradual loss of hearing (30 dB in 3 consecutive frequencies) over up to 72 hours in one or both ears. This can be a frightening experience for patients and should be treated as an emergency (1). The causes of ISSNHL are not fully understood, but various factors such as idiopathic, infectious, trauma, vascular, neoplastic, immunological, and ototoxic drugs have been suggested. However, the direct link between ISSNHL and these factors is still unknown. The incidence of ISSNHL is reported to be 5 to 20 per 100,000 per year. Around 30 to 65% of ISSNHL cases may resolve spontaneously. The prognosis of recovery depends on various factors, including the patient's age, the presence of vertigo at the time of onset, the degree and pattern of hearing loss, and the time between the onset of hearing loss and treatment (1,2). ISSNHL can also be related to factors such as atherosclerosis (3). As most cases are idiopathic, empiric treatment includes systemic and intratympanic injections of corticosteroids, antiviral and vasodilator agents, diuretics, and hyperbaric oxygen. However, due to the lack of complete knowledge, the treatment method is still controversial, and there is no strong evidence for any of the treatment options (1). Furthermore, patients who experience partial or no improvement in hearing or persistent tinnitus require ongoing monitoring of their hearing and psychological well-being. The severe consequences of delayed diagnosis and treatment, as well as the need for patients to be referred to various healthcare providers and the lack of randomized controlled trials to evaluate interventions, underscore the urgent need for evidence-based guidelines to aid clinicians in managing ISSNHL. Additionally, there are significant disparities in the assessment, treatment, counseling, and follow-up of ISSNHL patients worldwide (2,4). In Razavi Khorasan province, the main centers for treating patients with ISSNHL are the hospitals of Mashhad University of Medical Sciences. Patients in these hospitals are typically treated with a combination of systemic and intratympanic injections of corticosteroids. This study aims to investigate how this combined treatment affects hearing improvement and associated symptoms in patients with ISSNHL.

## Materials and Methods

*Patients*

This is a retrospective analysis of data prospectively collected in a clinical registry, Patients with ISSNHL who were referred from all medical centers in Razavi Khorasan province to the ISSNHL registry system from 2022-2024 were examined. ISSNHL was defined as the development of a 30 dB sensorineural hearing loss in at least three adjacent octave frequencies within a maximum period of 72 hours. After confirming the presence of idiopathic ISSNHL through hearing evaluations and obtaining informed consent, all subjects were treated with combined systemic and intratympanic corticosteroid injections. This study was approved by the ethics committee of Mashhad University of Medical Sciences (ethics code: IR.MUMS.REC.1400.145)."

*Procedure*

The initial evaluations included gathering demographic information (age and gender) and assessing symptoms associated with hearing loss through patient interviews. Following this, patients underwent hearing evaluations before receiving treatment. The hearing evaluation consisted of pure tone audiometry, speech audiometry, and tympanometry tests, which were conducted by an audiologist in a sound-treated room at the hearing clinic. The treatment involved a combination of systemic and intratympanic injections of corticosteroid. The intratympanic injection consisted of 8 mg/ml of dexamethasone with a dose of 0.4-0.8 ml in the anteroinferior part of the tympanic membrane, under microscopic view, until the middle ear cavity was filled. After the injection, the patient remained in a supine position for 15-30 minutes. According to Mashhad University protocol (5), a total of seven daily injections 7 daily injections were performed before conducting audiometry. If there was no improvement, the injection would be stopped at 7 days. If improvement continued, the injection would be continued with more intervals of up to 10 injections. The systemic medication included prednisolone tablets at a dose of 1 mg per kilogram of body weight, up to a maximum of 75 mg per day for one week, followed by tapering. The study exclusion criteria included the presence of a pathological lesion in the MRI of the base of the skull, bilateral cases of ISSNHL, lack of regular follow-up, the presence of diabetes, and concurrent neurological symptoms. Hearing evaluations were conducted again for all patients after the seventh injection and one month after the treatment, using the same conditions. The average of air conducted pure-tone hearing thresholds were measured at frequencies ranging from 250 to 8000 Hz. The initial grade of hearing and the extent of recovery were assessed using Modified Siegel's criteria (6). The alleviation of symptoms of tinnitus and vertigo was evaluated by asking questions to the patients.

*Adverse Event Monitoring*

at each visit patients were assessed for local complications (injection-site pain, tympanic membrane perforation, transient dizziness) via otoscopic examination and for systemic corticosteroid effects (blood pressure, blood glucose, weight changes, mood alterations) using standardized vital-sign checks and laboratory tests.

*Data analysis*

The study results were analyzed using SPSS software version 19. Descriptive statistics such as mean and standard deviation were used, and tables and graphs were also utilized to assess the recovery rate. The normal distribution was evaluated using a histogram and the Kolmogorov-Smirnov test. 

One-way ANOVA was employed for comparing groups with normally distributed data, while Kruskal-Wallis was used for variables such as the interval to the start of treatment, the degree of hearing loss on the seventh day, and the degree of hearing loss one month later, which did not follow a normal distribution.

## Results

In this study, 73 patients were treated. The age of the patients was 44.81±15.148 years, and 43.8% of them were female. The time interval between the onset of hearing loss and the start of treatment was 11.86±12.020 days. Other accompanying symptoms of the patients included dizziness in 4 (5.4%), vertigo in 33 (45.2%), ear fullness in 48 (65.7%), and tinnitus in 36 (49.3%). no local or systemic adverse events were observed during the treatment and follow-up period. The pre-treatment hearing grades of the patients is shown in Table 1.

**Table 1:** Levels of pre-treatment hearing grades based on Modified Siegel's criteria.

**Number and percentage of patients**	**Criteria**	**Grade**
4 (5.5%)	Average threshold value ≤25 dB HL	Grade 1
10 (13.7%)	Average threshold value 26-45 dB HL	Grade 2
34 (46.6%)	Average threshold value 46-75 dB HL	Grade 3
11 (15.1%)	Average threshold value 76-90 dB HL	Grade 4
14 (19.2%)	Average threshold value > 90 dB HL	Grade 5

The hearing grades was calculated across 250–8000 Hz based on Modified Siegel criteria, but the definition of ISSNHL is 30 dB in 3 consecutive frequencies. Therefore, patients in grade 1 may had Average threshold value lower than 25 dB HL.

All patients adhered to the established treatment regimen (Mashhad University protocol). Recovery outcomes for the patients are detailed in Table 2, highlighting hearing status after the seventh injection and one-month following treatment.

**Table 2:** The recovery rate of patients with ISSNHL after combined corticosteroid treatment.

**after one month**	**after seventh day**	**Criteria**	
22 (30.1%)	15 (20.5%)	The mean pure tone threshold is less than/equal to 25 dBHL	Complete recovery
10 (13.7%)	7 (9.6%)	The mean pure tone threshold is 26 to 45 dBHL and the improvement is more than 15 dB	partial recovery
12 (16.4%)	13 (17.8%)	The mean pure tone threshold is 46 to 75 dBHL and the improvement is more than 15 dB	Slight improvement
22 (30.1%)	30 (41.1%)	The mean pure tone threshold of 76 to 90 dBHL or an improvement of less than 15 dB	No improvement
7 (9.6%)	8 (11.0%)	The mean threshold of pure tone is greater than 90 dBHL	non-serviceable ear

**Figure 1:**
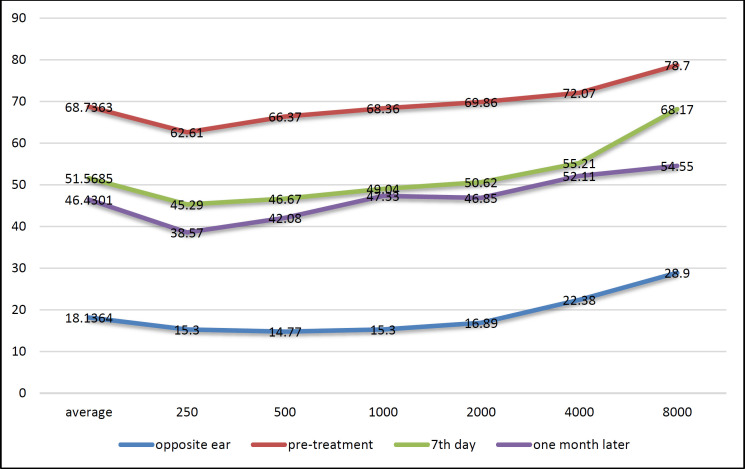
The mean changes in the thresholds of patients with ISSNHL at different frequencies

Figure 1 displays the average changes in the thresholds of patients with ISSNHL at different frequencies.

The comparison of hearing loss on the seventh day and one month later shows that the recovery process continues in some patients. The evaluations one month later confirm this trend. Table 3 compares the characteristics of patients in groups with different recovery rates.

**Table 3:** Comparison of different characteristics of ISSNHL patients with different rates of recovery.

**interval to the start of treatment**	**Pre-treatment hearing threshold average**	**Age**	**Sex**	**Group**	
7.44±6	47.92±22.363	39.93±15.746	F=8(53.3%) M=7(46.7%)	Complete recovery	the 7th day
7.43±3.7	69.59±29.053	44.8±18.161	F=2 (28.6%) M=5 (71.4%)	partial recovery	
11.50±9.5	90.38±17.349	46.54±12.183	F=9 (69.2%) M=4(30.8%)	Slight improvement	
15.50±14.6	58.72±20.717	46.34±14.341	F=11(36.7%) M=19(63.3%)	No improvement	
11.79±11	109.37±16.706	45.50±20.121	F=2 (25.0%) M=6 (75.0%)	non-serviceable ear	
0.3*	0<0.001*	0.7**		P value	
8.9±9.3	53.67±28.512	43.23±17.867	F=12(54.5%) M=10(45.5%)	Complete recovery	After one month
10.8±7.3	65.03±15.140	47.20±13.315	F=2 (20.0%) M=8(80.0%)	partial recovery	
10.91±11.9	97.37±22.900	43.25±13.844	F=9(75.0%) M=3(25.0%)	Slight improvement	
15.2±13.8	57.64±14.323	45.05±12.761	F=8(36.4%) M=14(63.6%)	No improvement	
13.2±10.9	107.14±16.707	48.29±19.998	F=1(14.3%) M=6(85.7%)	non-serviceable ear	
0.25*	<0.001*	0.9**		p-value	

*Kruskal-Wallis Test **One-way ANOVA

**Figure 2:**
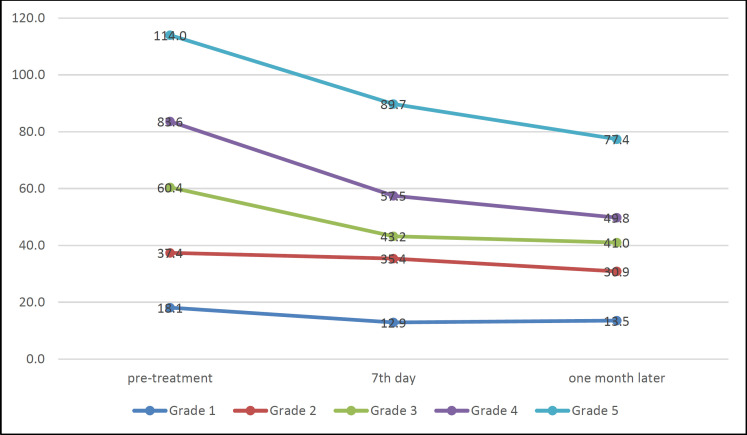
shows the pre-treatment and post-treatment thresholds for different grades.

The study results indicate a significant difference in pre-treatment hearing thresholds among various groups. Groups with better recovery show lower pre-treatment hearing thresholds. The only group that does not follow this pattern is the "No improvement" group, which, despite good pre-treatment hearing thresholds, has the highest interval to the start of treatment - more than 15 days. This delay may be a contributing factor to the lack of recovery in this group, and most of these patients have been treated as salvage. Figure 2- the pre-treatment and post-treatment thresholds for different grades of ISSNHL patients.

Based on the figure, the most significant changes after the seventh day are seen in patients with grades 4 and 5. Aside from improvements in hearing threshold, accompanying symptoms such as tinnitus and vertigo also showed improvement during the treatment. Out of the 36 patients with tinnitus, 9 (25.0%) fully recovered, 19 (52.8%) partially recovered, and 8 (22.2%) did not recover. All subjects with vertigo also experienced tinnitus, and there were no patients with vertigo but without tinnitus. Among the 33 people with vertigo, 22 patients (66.7%) fully recovered and 11 patients (33.3%) partially recovered. The different characteristics of these patients are detailed in Table 4.

**Table 4:** The relationship of tinnitus and vertigo with age, sex, degree of pre-treatment hearing thresholds, and hearing thresholds after seventh days and one month.

** P value**	**hearing thresholds**			**age**	**Sex**		**number**	**Symptom**	
	**one month later**	**seventh days**	**pre-treatment**		**male**	**Female**			
<0.001*	41.25±28.3	46.33±27.6	63 ±29.4	45.28±17.3	19(26.0%)	18(24.7%)	37(50.7%)	without	tinnitus
<0.001*	51.75±28.1	56.9±31.7	74.5±26.8	44.33±12.8	22(30.1%)	14(19.2%)	36(49.3%)	with	
	0.15***	0.1***	0.08**	0.7**	0.4****			P value	
<0.001*	41.6±27.9	46.33±27.6	62.28±28	45.26±16.8	22(24.7%)	18 (30%)	40(54%)	without	vertigo
<0.001*	52.2±28.7	57.8±32.3	76.4±27.1	44.27±13.1	19(26%)	14 (19%)	33(35%)	with	
	0.11***	0.1***	0.03**	0.7**	0.1****			P value	

*Friedman Test ** Independent T test ***Mann-Whitney Test ****Chi-square Tests

The results from Table 4 indicate that there was an improvement in hearing thresholds over the course of one month in both the groups with and without vertigo or tinnitus (P-value<0.001). Age and sex were found to not be determining factors for the presence of vertigo and tinnitus. Additionally, it was observed that pre-treatment hearing thresholds have a significant association with vertigo, but not with tinnitus. Furthermore, the hearing thresholds on the seventh day and one month later did not show any significant relationship with the presence of vertigo or tinnitus (Table 5).

This table indicate no correlation between vertigo/tinnitus recovery and pre/post-treatment hearing thresholds.

**Table 5:** the relationship between tinnitus and vertigo recovery with pre-treatment hearing thresholds, as well as hearing thresholds at seven days and one month later.

**one month later**	**seventh days**	**pre-treatment**	**Number (percent)**	**recovery**	
44.3±25	45.2±33.7	69.4±30.1	9(25%)	complete	tinnitus
47.9±22.8	55.4±29	74±26.6	19(53%)	partial	
69±38	73.6±32.1	81.4±25.4	8(22%)	No recovery	
0.2*	0.08*	0.5*		p-value	
45.3±20.4	51.2±28	73.1±29.6	22(66.7%)	complete	vertigo
66±38	71.1±37.1	83.1±21.1	11(33%)	partial	
0.1*	0.09*	0.2*		p-value	

*Kruskal-Wallis Test

## Discussion

This study aimed to investigate the effect of Combined Systemic and Intratympanic Corticosteroid Injections on hearing improvement and associated symptoms in patients with ISSNHL. The results of the study using Modified Siegel's criteria (6) showed complete, partial, and slight recovery in 30%, 14%, and 16% of patients, respectively, one month after treatment. The remaining patients did not experience proper recovery. However, the success of the treatment is not only related to hearing thresholds. Recovery of accompanying symptoms such as tinnitus and vertigo is also crucial for some patients. After one month, 25% and 53% of tinnitus cases and 67% and 33% of vertigo cases had complete and partial recovery, respectively, indicating a high percentage of treated patients, especially in cases of vertigo. The presence of accompanying symptoms such as vertigo is associated with a worse recovery prognosis than cases without vertigo (7). This trend was also observed in our study, but this difference was not statistically significant.

In general, there are various treatment methods suggested for ISSNHL patients. Systemic corticosteroid treatment is very common, but its results and treatment value are not well known (8,9). The therapeutic results of intratympanic corticosteroid injections are similar to systemic corticosteroids (8,10,11). However, a review article reported better threshold improvement with intratympanic injection (12). For ISSNHL patients with vertigo, intratympanic injection may have better therapeutic effects (7). Similar results or better therapeutic effects are observed in combined treatments (12,13). In our previous study, systemic steroid treatment along with intratympanic dexamethasone injections showed complete, partial, and slight improvement in 15%, 15%, and 34% of patients, respectively (5).

In the present study, under this systematic monitoring protocol, no patients experienced any side effects from the treatment. While various studies have mentioned different side effects associated with intratympanic injection, in many cases these side effects are mild. Some studies have even reported no serious side effects (14). The most important factor influencing recovery in this study was the degree of pre-treatment hearing loss. Less recovery was observed in cases with a longer interval between the onset of symptoms and the start of treatment. Since spontaneous recovery can occur in cases of ISSNHL, the prognosis for treatment may worsen over time, and poorer treatment outcomes may become more probable (9). Also, because the treatment window overlaps with the period of highest spontaneous recovery, the recovery rate may reflect natural recovery, treatment effect, or both. The main limitation of our study was the inability to conduct long-term follow-ups exceeding one month. Many patients did not cooperate with re-evaluation after one month, making it impossible to track further improvements in hearing over time. Additionally, we had a wide range of ages among the patients, and with an age increase of over 50 years, there is a possibility of the aging effects on the treatment results. However, combined corticosteroid treatment improved the hearing thresholds in sixty percent of patients and resolved the tinnitus and vertigo in most patients. The combined systemic and intratympanic corticosteroid injections may be considered as an effective treatment option for ISSNHL, but randomized controlled trials are required to establish whether it should be recommended as first-line therapy.

For future studies, we recommend prospective, randomized placebo-controlled trials to distinguish true therapeutic benefit from the natural course of ISSNHL. This is the first study based on the information registered in the ISSNHL registry system of Khorasan Razavi-Iran. Our plan for the future is to design more extensive studies, including multicenter randomized clinical trials, to compare different treatment methods.

## Conclusion

The combined systemic and intratympanic corticosteroid therapy was linked to clinical improvements in about 60 % of patients, reducing hearing loss, tinnitus, and vertigo.

## References

[1] Flint PW HB, ‎ Lund VJ, ‎ Niparko JK, ‎ Robbins KT, Thomas JR, ‎ et al (2020). Cummings Otolaryngology-Head and Neck Surgery.

[2] Stachler RJ, Chandrasekhar SS, Archer SM, Rosenfeld RM, Schwartz SR, Barrs DM (2012). Clinical practice guideline: sudden hearing loss. Otolaryngology--head and neck surgery : official journal of American Academy of Otolaryngology-Head and Neck Surgery..

[3] Rajati M, Azarpajooh MR, Mouhebati M, Nasrollahi M, Salehi M, Khadivi E (2016). Is Sudden Hearing Loss Associated with Atherosclerosis?. Iranian journal of otorhinolaryngology..

[4] Chau JK, Lin JR, Atashband S, Irvine RA, Westerberg BD (2010). Systematic review of the evidence for the etiology of adult sudden sensorineural hearing loss. The Laryngoscope..

[5] Rajati M, Ghasemi MM, Sharifian MR, Nourizadeh N, Yousefi R, Hosseinpoor M (2022). Intratympanic Steroid for the Management of Sudden Hearing Loss: Introduction of a Tapering Method. Iranian journal of otorhinolaryngology..

[6] Cheng YF, Chu YC, Tu TY, Shiao AS, Wu SL, iao WH (2018). Modified Siegel's criteria for sudden sensorineural hearing loss: Reporting recovery outcomes with matched pretreatment hearing grades. Journal of the Chinese Medical Association : JCMA..

[7] Yu H, Li H (2018). Association of Vertigo With Hearing Outcomes in Patients With Sudden Sensorineural Hearing Loss: A Systematic Review and Meta-analysis. JAMA otolaryngology-- head & neck surgery..

[8] Plontke SK, Meisner C, Agrawal S, Cayé-Thomasen P, Galbraith K, Mikulec AA (2022). Intratympanic corticosteroids for sudden sensorineural hearing loss. The Cochrane database of systematic reviews..

[9] Wei BP, Stathopoulos D, O'Leary S (2013). Steroids for idiopathic sudden sensorineural hearing loss. The Cochrane database of systematic reviews..

[10] Sialakis C, Iliadis C, Frantzana A, Ouzounakis P, Kourkouta L (2022). Intratympanic Versus Systemic Steroid Therapy for Idiopathic Sudden Hearing Loss: A Systematic Review and Meta-Analysis. Cureus..

[11] Mirian C, Ovesen T (2020). Intratympanic vs Systemic Corticosteroids in First-line Treatment of Idiopathic Sudden Sensorineural Hearing Loss: A Systematic Review and Meta-analysis. JAMA otolaryngology-- head & neck surgery..

[12] Li J, Ding L (2020). Effectiveness of Steroid Treatment for Sudden Sensorineural Hearing Loss: A Meta-analysis of Randomized Controlled Trials. The Annals of pharmacotherapy..

[13] Nourizadeh N, Rezaiee N, Rajati M, Dabiri S, Afzalzadeh MR, Hasanabadi K (2023). Evaluation of Sudden Sensory-Neural Hearing Loss Patients Treated with Systemic Steroids with Additional Intratympanic Dexamethasone Injection in Different Intervals; a Clinical Trial Study. Indian journal of otolaryngology and head and neck surgery : official publication of the Association of Otolaryngologists of India..

[14] Devantier L, Callesen HE, Jensen LR, Mirian C, Ovesen T (2022). Intratympanic corticosteroid as salvage therapy in treatment of idiopathic sudden sensorineural hearing loss: A systematic review and meta-analysis. Heliyon..

